# Low expression of IGFBP4 and TAGLN accelerate the poor overall survival of osteosarcoma

**DOI:** 10.1038/s41598-022-13163-8

**Published:** 2022-06-03

**Authors:** Yue Xi, Jianlin Liu, Gufeng Shen

**Affiliations:** Department of Orthopaedics, Shanghai Fourth People’s Hospital Affiliated to Tongji University School of Medicine, 1279 Sanmen Road, Hongkou District, Shanghai, 200011 China

**Keywords:** Medical research, Oncology

## Abstract

Osteosarcoma is a common malignant bone tumor characterized by the production of osteoid stroma by the tumor. However, effect of IGFBP4 and TAGLN on the survival of osteosarcoma is unclear. The GEO database was used to identify the differentially expressed genes (DEGs) between control samples and osteosarcoma. Genes for biological process (BP), cellular composition (CC), and molecular function (MF) were examined using DAVID, Metascape, and GSEA. GSE14359 and GSE36001 were downloaded in the GEO database. GEO2R was used to find DEGs between control samples and osteosarcoma. The cytoHubb also found the hub genes of IGFBP4 and TAGLN. The Kaplan–Meier method was used to analyze overall survival. A total of 134 patients with osteosarcoma were enrolled in this study. The RNA levels of IGFBP4 and TAGLN were evaluated by RT-qPCR. The correlation between IGFBP4 and TAGLN expression and their associations with clinical indicators were analyzed using Spearman's rho test and Pearson's Chi-squared test. Univariate and multivariate Cox regression analyses were used to determine the potential prognostic factors. And the animal model was used to verify the role of hub genes on the osteosarcoma by the RT-qPCR and immunofluorescence. Support Vector Machine (SVM) was performed to construct the correlation among the expression of IGFBP4, TAGLN, and osteosarcoma. Through bioinformatics, IGFBP4 and TAGLN were identified as the hub genes of osteosarcoma. And osteosarcoma patients with high expression levels of IGFBP4 (HR = 0.56, P = 0.013) and TAGLN (HR = 0.52, P = 0.012) had better overall survival times than those with low expression levels. The results showed that pathologic grade (P = 0.017), tumor metastasis (P < 0.001), and enneking stage (P < 0.001) were significantly correlated with IGFBP4. Also, pathologic grade (P = 0.002), tumor metastasis (P < 0.001), and enneking stage (P < 0.001) were significantly related to the TAGLN. Spearman’s correlation coefficient displayed that IGFBP4 were significantly correlated with the tumor metastasis (ρ = − 0.843, P < 0.001), enneking stage (ρ = − 0.500, P < 0.001), and TAGLN (ρ = 0.821, P < 0.001). IGFBP4 (HR = 0.252, 95% CI 0.122–0.517, P < 0.001) and TAGLN (HR = 0.155, 95% CI 0.089–0.269, P < 0.001) were significantly associated with overall survival. Based on the qPCR and immunofluorescence, IGFBP4 and TAGLN were down-regulated in the osteosarcoma tissue than the control group. And the SVM presented that there exists strong relationship among the expression of IGFBP4, TAGLN, and osteosarcoma. IGFBP4 and TAGLN may be attractive molecular targets for osteosarcoma, opening a new avenue for research into the disease.

## Introduction

Osteosarcoma is a common malignant bone tumor characterized by the production of osteoid stroma. It is most common in adolescents and can present as bone and joint pain and local lumps^1^. At the first visit, almost 15% of osteosarcoma patients were found to have distant metastases^2^. The 5-year survival rate for patients with early metastasis is less than 20%^3,4^. In modern medicine, the etiology of osteosarcoma and the mechanism of abnormal cell proliferation are still unknown. As a result, research into the molecular mechanism of osteosarcoma is critical, as is the development of new therapeutic drugs. IGFBP4 gene is located at 17q12-q21.1, with a length of 2 246 bp. It encodes human IGF-BP4 protein containing 237 amino acid residues and 20 cysteine residues^5^. IGFBP4 (insulin-like growth factor-binding protein 4) is a secreted protein expressed in various normal tissues but has low expression in most tumors, such as liver cancer^6^, gastric cancer^7^, breast cancer^8^, etc. IGFBP4 could bind to IGFs and regulate their biological effects^9^. Mohan et al. initially demonstrated that IGFBP4 inhibited bone growth by inhibiting IGF1 in a dose-dependent manner^10^. In transgenic mice overexpressing IGFBP4, the number of osteoblasts and bone formation efficiency was significantly inhibited^11^. In addition, IGFBP4 might play an essential role in bone development and differentiation. IGFBP4 was discovered to be substantially expressed in the condylar cartilage disc system, and the subarticular cavity of mice, but IGFBP4 expression in the fibrous interstitial tissue of the subarticular cavity was significantly decreased, according to Shibata^12^.

TAGLN gene is located on 11q23.2. The gene is 5.4 kb in length and consists of 5 exons and 4 introns. The mRNA is 1556 bp in length, a member of the filaments family^13^. Smooth muscle protein 22 (SM22), encoded by the TAGLN gene, is a stress fiber-related protein that regulates cell growth and contraction by stabilizing actin filaments. Nuclear connection factors are found in the promoter of TAGLN, which influence the expression of related genes in smooth muscle tissue and may be involved in epithelial interstitial transformation transcriptional regulation^14^. Relevant study show that TAGLN may be closely related to the canceration or migration and diffusion of tumors and is abnormally expressed in many tumor diseases^15^. TAGLN could play an essential role in the biological processes of tumor cell proliferation, apoptosis, migration, invasion, and metastasis^16^. TAGLN protein is mainly located in the cytoplasm and nucleus and can be regulated by post-translational modifications such as phosphorylation, acetylation, ubiquitination, and methylation. Overexpression of TAGLN in human cell line MDA-MB-231 can inhibit cell migration and invasion in vitro^17^. However, the molecular mechanism of IGFBP4 and TAGLN on osteosarcoma has not been further explored in these studies.

Bioinformatics studies biological problems using the methods of applied mathematics, informatics, statistics, and computer science. Some studies have researched the expression profile of RNA in osteosarcoma by using bioinformatics methods and found that bioinformatics analysis might be one valuable tool for the research of osteosarcoma^18,19^.

Therefore, bioinformatics technology was used to excavate the hub genes of osteosarcoma for enrichment analysis, pathway analysis, and survival analysis. Use public data to verify the role of hub genes in osteosarcoma. In the current study, we evaluated the expression pattern of IGFBP4 and TAGLN in patient-derived osteosarcoma tissues. We also explored the clinical implications of IGFBP4 and TAGLN expression status in patients with osteosarcoma, and the animal experiment was performed to verify the role of IGFBP4 and TAGLN on the osteosarcoma.

## Materials and methods

### Public dataset

We downloaded GSE14359 and GSE36001 from the GEO database (https://www.ncbi.nlm.nih.gov/geo/). The GSE14359 has 18 osteosarcoma tissue samples and 2 non-neoplastic primary human osteoblasts, whereas the GSE36001 contains 19 osteosarcoma tissue samples and 2 non-neoplastic primary human osteoblasts.

### DEGs identification

We applied GEO2R (http://www.ncbi.nlm.nih.gov/geo/geo2r). GEO2R is an interactive web tool. It allows the user to compare the two groups of GEO series or more than two sets of samples to identify the different genes expressed in different experimental conditions. The results show a list of genes in order of importance. GEO2R uses the GEOquery and Limma R packages from the Bioconductor project to perform comparisons against the processed data tables provided by the original submitter. The cut-off criteria were that a log (FC) > 1 or log (FC) < − 1 and P-value < 0.05.

### DEGs annotation

The Database for Annotation, Visualization and Integrated Discovery (DAVID) (https://david.ncifcrf.gov/home.jsp), Metascape (http://metascape.org/gp/index.html) are two powerful annotation tools that can perform the biological process (BP), cellular component (CC), molecular function (MF) analysis on genes. We annotated the function of common DEGs through DAVID and Metascape.

### Protein–protein interaction (PPI) network construction

The Search Tool for the Retrieval of Interacting Genes (STRING) (http://string-db.org) can convert DEGs into expressed proteins and structure the PPI network. We got a PPI network of common DEGs through STRING and visualized it by Cytoscape (version 3.8.0).

### Hub genes identification and expression

Molecular Complex Detection tool (MCODE) (version 1.6.1), an open plug-in of Cytoscape, was performed to identify system modules from the PPI network. The criteria were that the MCODE scores > 5, maximum depth = 100, cut-off = 2, k-score = 2, and node score cut-off = 0.2. In addition, we also used cytoHubb to screen out hub genes and sequenced them by two different arithmetics of MCC and DMNC.

### Overall survival analysis of hub genes

The effect of hub gene expression on osteosarcoma survival was investigated. The database sources included GEO, EGA, and TCGA. The tool's primary purpose is a meta-analysis based on discovering and validating survival biomarkers.

### The Comparative Toxicogenomics Database (CTD)

CTD provides manual management information on chemical-gene/protein interactions, chemical–disease, and gene–disease relationships. These data, combined with functional and pathway data, help to develop hypotheses about the mechanisms. There are other ongoing projects, including manual processing of exposure data and chemical–phenotypic relationships to help identify predisease biomarkers.

### Gene set enrichment analysis (GSEA)

The basic idea of Gene Set Enrichment Analysis (GSEA) is to use a predefined set of genes to sequence genes according to their degree of differential expression in two types of samples. Then it could check to see if the set of genes is enriched at the top or bottom of the sequencing list. Gene collection enrichment analysis detects changes in expression of collections of genes rather than individual genes, and therefore can include these subtle changes in expression and expect better results.

### Human Protein Atlas for the protein expression of the IGFBP4 and TAGLN1

The Human Protein Atlas (HPA) provides tissue and cellular distribution information for 26,000 Human proteins. In this database, the expression of each protein in 64 cell lines, 48 human normal tissues and 20 tumor tissues was examined in detail using immunoassay techniques (western blotting, immunofluorescence and immunohistochemistry) using highly specific antibodies.

### Patients and ethics

The object of the study was 134 patients who underwent surgery in Shanghai Fourth people's Hospital Affiliated with Tongji University School of Medicine between January 2018 and December 2019. Inclusion criteria: patients with pathologic diagnosis of osteosarcoma, patients without a surgical history. Exclusion criteria: patients with poor cardiac function, pulmonary function, and liver and kidney function who could not tolerate surgery, patients requiring emergency surgery.

The study was confirmed by the Ethics Committee of Shanghai Fourth people's Hospital Affiliated to Tongji University School of Medicine. Written informed consent was obtained from all patients. All methods were performed following the relevant guidelines and regulations. This is a biospecimen protocol, and it is retrospectively analyzed.

### Diagnosis of tumor metastasis from osteosarcoma

Patients had pathologically confirmed osteosarcoma. Osteosarcoma tissue was taken from osteosarcoma patients, and all samples were stored at − 80 °C until RNA separation.

### Clinical characteristic index

Clinical characteristics include sex, age, tumor size, pathologic grade, tumor metastasis, enneking stage, the expression of IGFBP4 and TAGLN, overall survival time.

### Definition of the low, intermediate, and high expression

The followed criterion defined low, medium, and high expression of IGFBP4. All the individuals’ IGFBP4 expressions were divided by the quartile method. Low expression of IGFBP4: relative mRNA expression < 25%; Moderate expression of IGFBP4: 25% ≤ relative mRNA expression ≤ 75%; High expression of IGFBP4: relative mRNA expression > 75%.

Low, intermediate, and high expression of TAGLN was defined by the followed criterion. All the individuals’ TAGLN expressions were divided by the quartile method. Low expression of TAGLN: relative mRNA expression < 25%; Moderate expression of TAGLN: 25% ≤ relative mRNA expression ≤ 75%; High expression of TAGLN: relative mRNA expression > 75%.

### Animal model of osteosarcoma

The C57BL/6 mice (male, 8 ± 1 weeks) were weighed, and this information was recorded. They were then numbered and assigned to groups (normal: n = 15; osteosarcoma: n = 15) according to the random number table method. The salt solution of radionuclide was injected into the mice in the osteosarcoma group (subcutaneous under the right lateral axilla behind the skin) and local massage was given at the injection site daily to induce osteosarcoma formation.

### RT-qPCR

Tumor tissues of osteosarcoma patients were obtained via surgery and preserved at − 80 °C immediately. The traditional Trizol extraction techniques for RNA separation include liquid nitrogen grinding, Trizol cracking, chloroform extraction, centrifugation, chloropropanol precipitation, alcohol washing, and centrifugation. (1) Put the abrasive tissue fluid on ice, add Trizol to crack for 5–10 min, blow it gently with the head of a gun and then drain the liquid into the imported EP tube. (2) Add 1/5 volume of chloroform, mix the liquid up and down and let sit at 4 °C for 10–15 min. (3) Centrifugation at 4 °C for 15 min, be sure to choose low-temperature centrifugation. After centrifugation, the EP tube is divided into three layers, and RNA is in the supernatant. The EP tube is gently removed from the centrifuge to avoid the material's shock in the tube, causing the lower layer to precipitate. When absorbing supernatant, be sure to act gently and avoid absorbing too much. Generally, absorb 400–500 μL to avoid absorbing the lower layer of precipitation. Put the liquid in a new EP tube. (4) Add isopropyl alcohol in equal volume, stand at 4 °C for 10 min, then centrifuge at 12,000 rpm for 10 min. Isopropyl alcohol is mainly used to precipitate RNA. (5) After the EP tube was removed, the sidewall precipitation could be seen, and the supernatant was gently aspirated and discarded. (6) Add 75% alcohol into the EP tube to wash the precipitate to help separate the remaining organic reagent, gently tap the precipitate to float in the alcohol, let the alcohol fully contact the precipitate, and fully dissolve the organic reagent, then centrifuge at 4 °C for 5 min. Gently blot and discard the supernatant. (7) Put the EP tube into the centrifuge again for quick centrifugation and discard the residual liquid in the tube wall. Then the EP tube was placed in a super-clean table to dry for 5–10 min. Note that THE RNA sample should not be too dry, or it will be difficult to dissolve. (8) Add 50 μL DEPC treatment water and shake to dissolve the precipitation. Measure the RNA concentration. The RNA is stored at − 80 °C. RT-qPCR was performed on a Roche Light Cycler 480 instrument (Roche, Basel, Switzerland) using 2 × SG Fast qPCR Master Mix (Sangon, Shanghai, China). After thawing, mix and briefly centrifuge the components of the kit. Store on ice. Add the following reagents into a sterile, nuclease-free tube on ice in the indicated order: Template RNA 2 µg, Primer Oligo (dT) 18 primer 0.5 µL and Random Hexamer primer 0.5 µL or gene-specific primer 1 µL, 5 × Reaction Buffer 4 µL, Servicebio^®^RT Enzyme Mix^a^ 1 µL, Water, nuclease-free to 20 µL, Total volume 20 µL. Mix gently and centrifuge briefly. Incubate for 5 min at 25 °C, 30 min at 42 °C. Terminate the reaction by heating at 85 °C for 5 s. PCR amplification included pre-denaturation (95 °C, 10 min), cycle (40 times, 95 °C, 15 s to 60 °C, 60 s), Melt Curve (60–95 °C, 0.3 °C/15 s). All experiments were repeated twice, CT-values were pre-converted into relative quantities (Q) using the equation Q = 2^−ΔΔCT^ for subsequent statistical analysis. This study was done regarding MIQE recommendation 23. The expression of IGFBP4 and TAGLN was detected by RT-qPCR^18,20^. GAPDH was used as the control gene.

The primers of the genes were as followed [Sequence (5′–3′)]:

- IGFBP4-hF: AGCAGTATTGGTGCCTCG;
- IGFBP4-hR: CCTGTTCTTCCCATGTTGA;
- TAGLN-hF: AGGTCTGGCTGAAGAATGG;
- TAGLN-hR: TGTGAGCGTGGCGTGTTA;
- LYN-hF: GTGTACGGATCAAGTCAG;
- LYN-hR: GAGATGCTACAACAGGGA;
- TNC-hF: TCCCAAACACCATCCTAC;
- TNC-hR: GAACAAATCCTAGACCCT;
- TGFB2-hF: ACGCACAGTCTCAAATAGC;
- TGFB2-hR: AGCCAGCAAGAAACCAAG;
- IGFBP1-hF: AGAGGGAGCACATCACAT;
- IGFBP1-hR: TGGAGTGGACCTTCTTTG;
- SERPINE1-hF: AATACTTAGCATAGCCATCA;
- SERPINE1-hR: TACAGTCATAAGCCACCTT;
- ANPEP-hF: AACACCCTCTTCCTGATTG;
- ANPEP-hR: AGTCTCCGCGACCTTTAT;
- GAPDH-hF: AGTCCACTGGCGTCTTCA;
- GAPDH-hR: GAGCGTGTCCATAGGGTG.

### Immunofluorescence assay for IGFBP4 and TAGLN1

Washing three times with phosphate buffered saline (PBS) (pH7.4) (5 min/time), immersed the osteosarcoma sections in EDTA antigen retrieval buffer (pH8.0) (Servicebio G1206, Wuhan, China) to make antigen retrieval. Treat with PBS(PH7.4) (three times, 5 min/time), adding 3% BSA (Servicebio, G5001, Wuhan, China) to block non-specific binding for 30 min. Throwing away the blocking solution, sections are incubated by IGFBP4 antibody (dilution rate = 1:400, bs-10585R, BIOSS, Beijing, China) (overnight, at 4 °C). Washing the sections again with PBS (pH 7.4), fluorescent secondary antibodies (dilution rate = 1:5000) responding to the primary antibodies were added (room temperature, 50 min, dark condition). Then incubated with DAPI solution (Servicebio, G1012, Wuhan, China) (room temperature, 10 min, darkness) to counterstaining nucleus. Finally, using spontaneous fluorescence quenching reagent (5 min) (Servicebio, G1221, Wuhan, China) to make spontaneous fluorescence quenching and sealing the sections with anti-fade mounting medium. The detecting process of TAGLN1 was the same as above using TAGLN1 antibody (dilution rate = 1:400, bs-2178R, BIOSS, Beijing, China). Fluorescence microscopy (Nikon NIKON ECLIPSE C1) showed that the nuclei were blue (excitation wavelength 330–380 nm and emission 420 nm) and the positive expression was red or green (FITC glows green by excitation wavelength 465–495 nm and emission 515–555 nm; CY3 glows red by excitation wavelength 510–560 nm and emission 590 nm).

### Support vector machine (SVM)

Support vector machine (SVM) is a kind of generalized linear classifier that classifies data by supervised learning method. The decision boundary is the maximum-margin hyperplane solved for the learning sample. SVM uses Hinge loss function to calculate empirical risk and adds regularization term to the solving system to optimize structural risk. It is a classifier with sparsity and robustness. SVM, one of the common kernel learning methods, can be used for nonlinear classification by kernel method. In this study, the input value of SVM is the relative expression value of IGFBP4 and TAGLN, and the output variable is the tumor size of osteosarcoma.

### Statistical analysis

The data statistics are expressed as sample size and percentage of the total. The Pearson Chi-square test analyzed the relationship between IGFBP4, TAGLN, and related clinical factors. Spearman-rho correlation test was used to test the correlation between the two further. The statistical results used univariate and multivariate cox regression analyses to calculate each variable's hazard ratios (HRs). Finally, we used the Kaplan–Meier method to explore OS.

All statistical analyses were conducted using SPSS software, version 24.0 (IBM Corp., Armonk, NY, USA). P < 0.05 was considered statistically significant.

### Ethics approval and consent to participate

All experiments were approved by the Ethics Committee of Shanghai Fourth people's Hospital Affiliated with Tongji University School of Medicine. All research was performed following relevant guidelines/regulations, and informed consent was obtained from all participants and/or their legal guardians.

## Results

### Identification of DEGs

**Figure 1:**
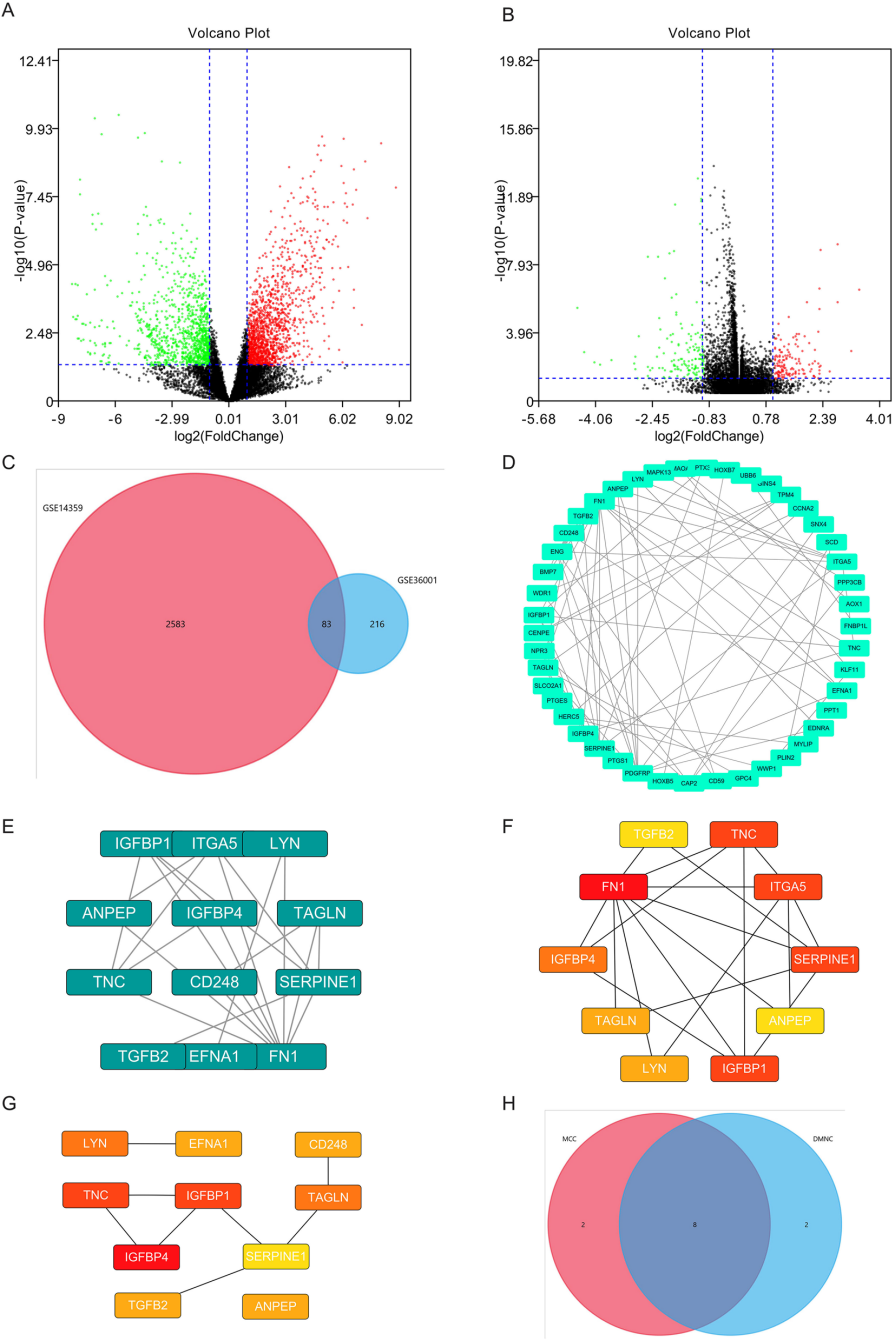
Two the DEGs. (**A**) The volcano plots present DEGs in the GSE14359. (**B**) The volcano plots present DEGs in the GSE36001. (**C**) The common DEGs between GSE14359 and GSE36001. (**D**) PPI network. (**E**) The key module of MCODE analysis. (**F**) The top 10 genes screened by cytoHubb in MCC. (**G**) The top 10 genes screened by cytoHubb in DMNC. (**H**) Venn diagram figured out eight mutual genes between the algorithms, including IGFBP4, TAGLN, LYN, TNC, TGFB2, IGFBP1, SERPINE1, ANPEP.

One volcano plot presented the DEGs between osteosarcoma and control samples in the GSE14359 (Fig. 1A), and the other volcano plot presented the DEGs between osteosarcoma and control samples in the GSE36001 (Fig. 1B). The Venn diagram showed 83 DEGs shared between the two datasets (Fig. 1C).

### Protein–protein interaction network and hub genes

PPI is shown in Fig. 1D. The key module of MCODE analysis was conducted (Fig. 1E). The top 10 genes screened by cytoHubb in two algorithms were established (Fig. 1F,G), and the Venn diagram figured out 8 mutual genes between the algorithms, which included IGFBP4, TAGLN, LYN, TNC, TGFB2, IGFBP1, SERPINE1, ANPEP (Fig. 1H).

### DEGs annotation

**Figure 2:**
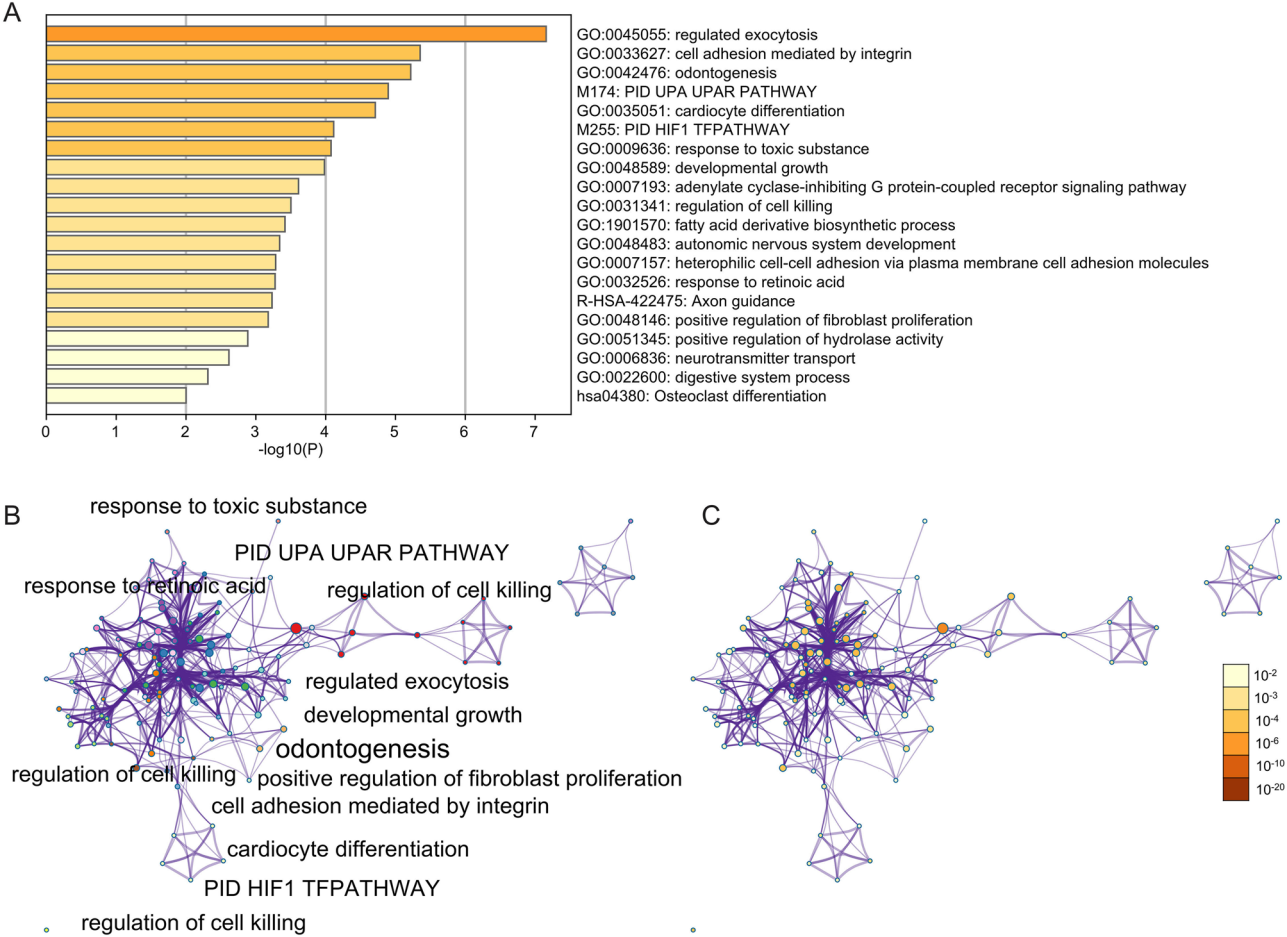
Enrichment analysis for the DEGs by Metascape. (**A**) Heatmap of enriched terms across input differently expressed gene lists, colored by P-values, via the Metascape. (**B**) Network of enriched terms colored by cluster identity, where nodes that share the same cluster identity are typically close. (**C**) Network of enriched terms colored by P-value, where terms containing more genes tend to have a more significant P-value.

**Figure 3:**
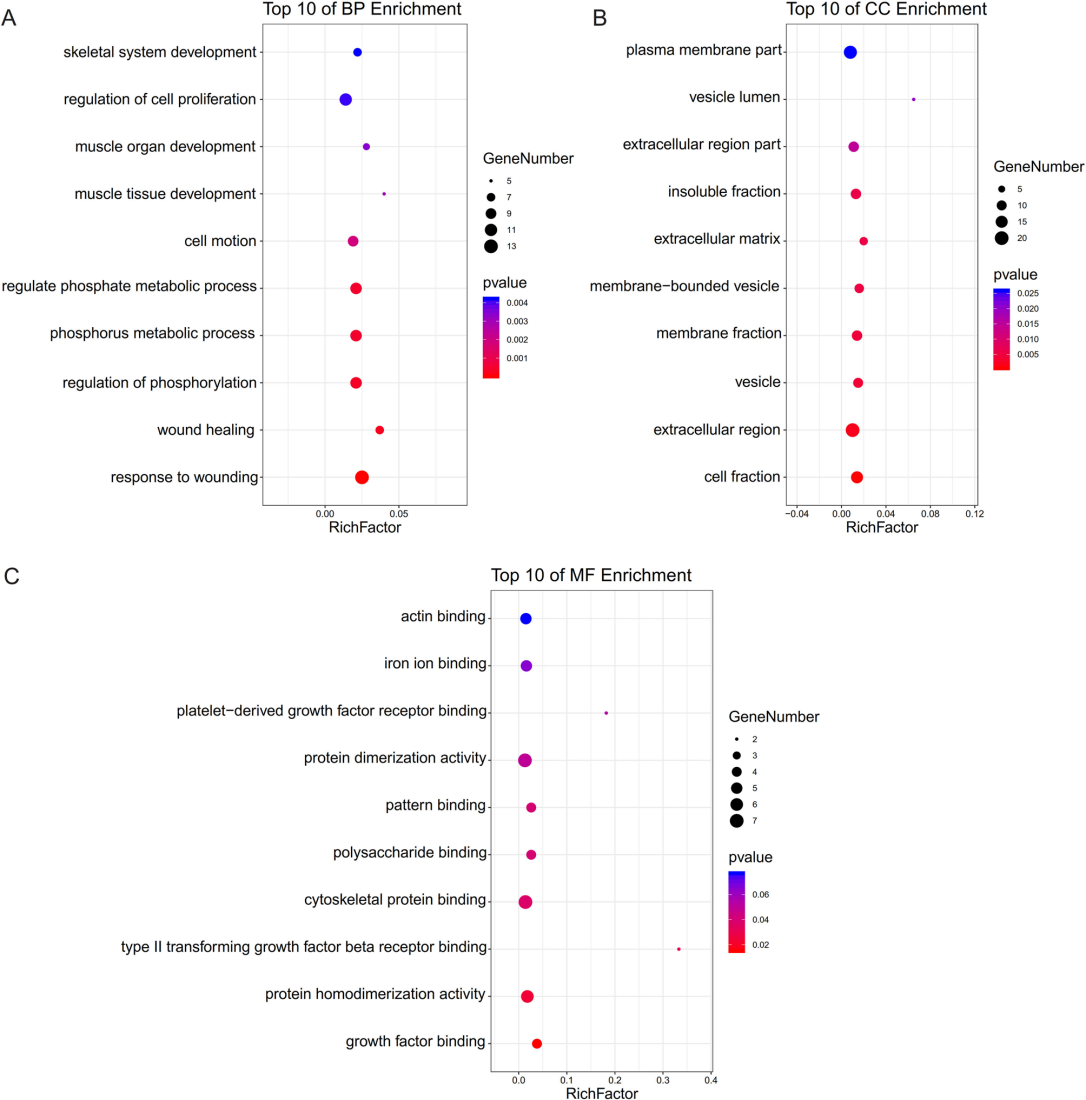
The functional annotation for the DEGs based on the DAVID. (**A**) BP, (**B**) CC, (**C**) MF.

Enrichment analysis by Metascape is displayed in Fig. 2. Bubble diagrams show DEGs associated with BP, CC, and MF (Fig. 3). DEGs associated with BP were primarily enriched in skeletal system development, cell proliferation regulation, muscle organ development, muscle tissue development, cell motion, phosphate metabolic process regulation, phosphate metabolic process rule, phosphorylation regulation, wound healing and wound response (Fig. 3A). The variations in DEGs linked with CC were mainly enriched in plasma membrane part, vesicle lumen, extracellular region part, insoluble fraction, extracellular matrix, membrane-bounded vesicle, membrane fraction, and vesicle cell fraction (Fig. 3B). Actin binding, iron ion binding, platelet-derived growth factor receptor binding, protein dimerization activity, pattern binding, polysaccharide binding, cytoskeletal protein binding, type II transforming growth factor-beta receptor binding, protein homodimerization activity, and growth factor binding were the most common variations in DEGs associated with MF (Fig. 3C).

### The expression of hub genes

**Figure 4:**
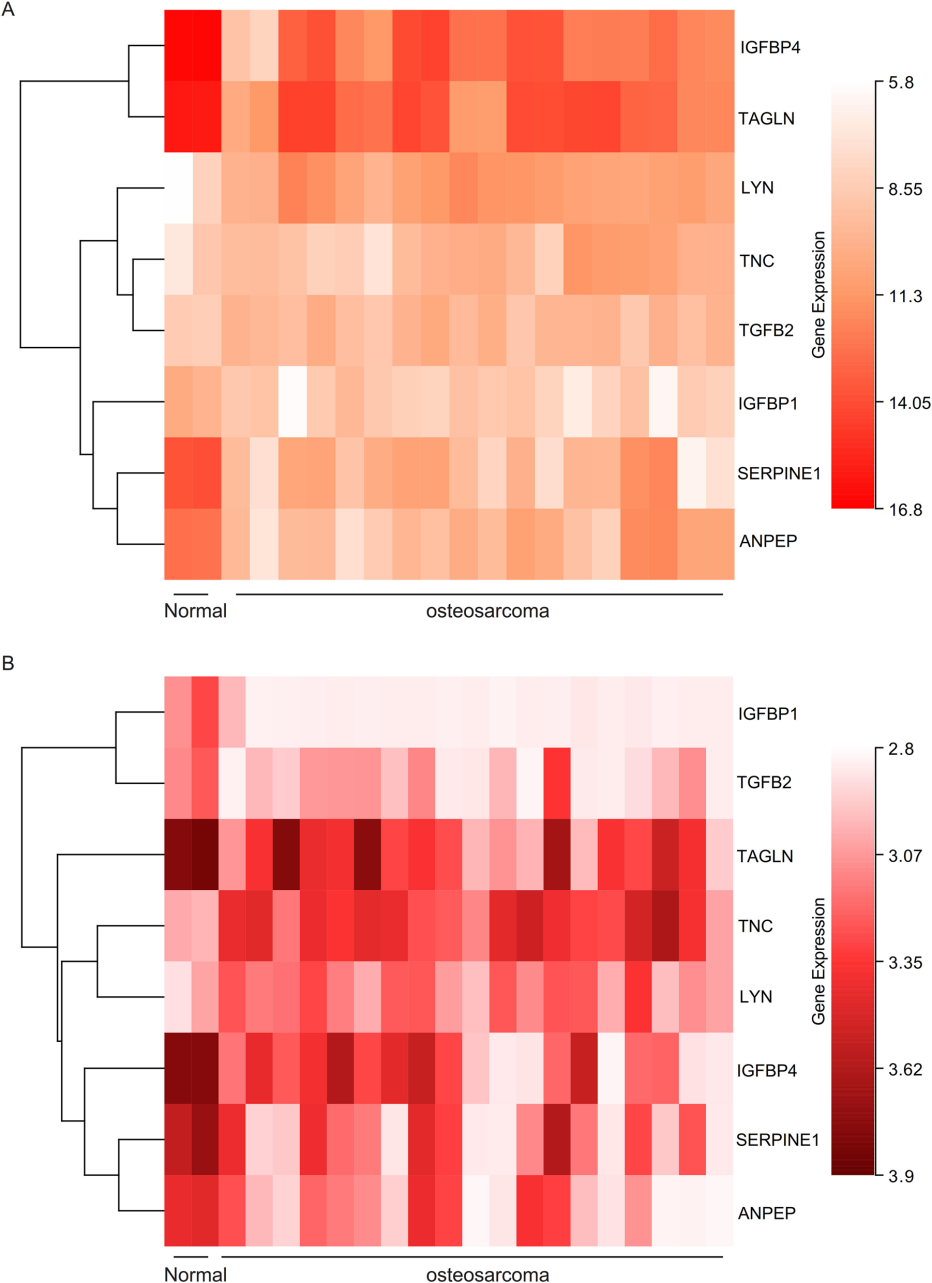
The expression analysis for the hub genes. (**A**) The heat map showed the expressions of the hub genes in GSE14359. (**B**) The heat map showed the expressions of the hub genes in GSE36001.

The expressions of the hub genes in GSE14359 (Fig. 4A) and GSE36001 (Fig. 4B) are shown in the two heat maps. And LYN, TNC, TGFB2, IGFBP1were up-regulated in the osteosarcoma tissue samples, while IGFBP4, TAGLN, SERPINE1, ANPEP were down-regulated.

### Effect of hub genes on the overall survival of osteosarcoma

**Figure 5:**
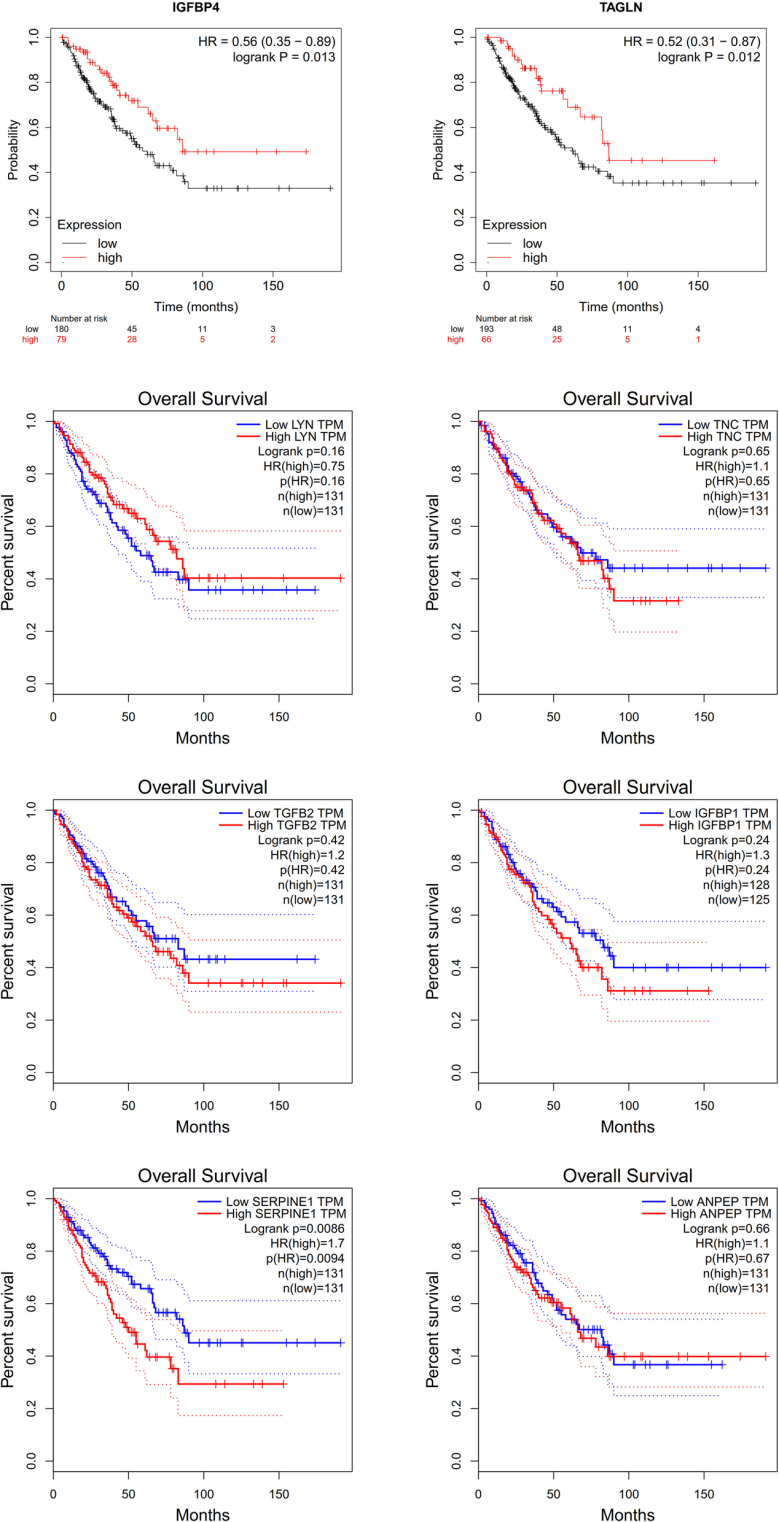
Effect of hub genes on the overall survival of osteosarcoma.

The expression of LYN, TNC, TGFB2, IGFBP1, and ANPEP (P > 0.05) had no significant effect on the overall survival of osteosarcoma. Osteosarcoma patients with high expression levels of IGFBP4 (HR = 0.56, P = 0.013) and TAGLN (HR = 0.52, P = 0.012) had better general survival times than those with low expression levels. However, osteosarcoma patients with high expression levels of SERPINE1 (HR = 1.7, P = 0.0094) had poorer overall survival times than those with low expression levels (Fig. 5).

### Identification of inference score of hub genes in the osteosarcoma by the CTD database

**Figure 6:**
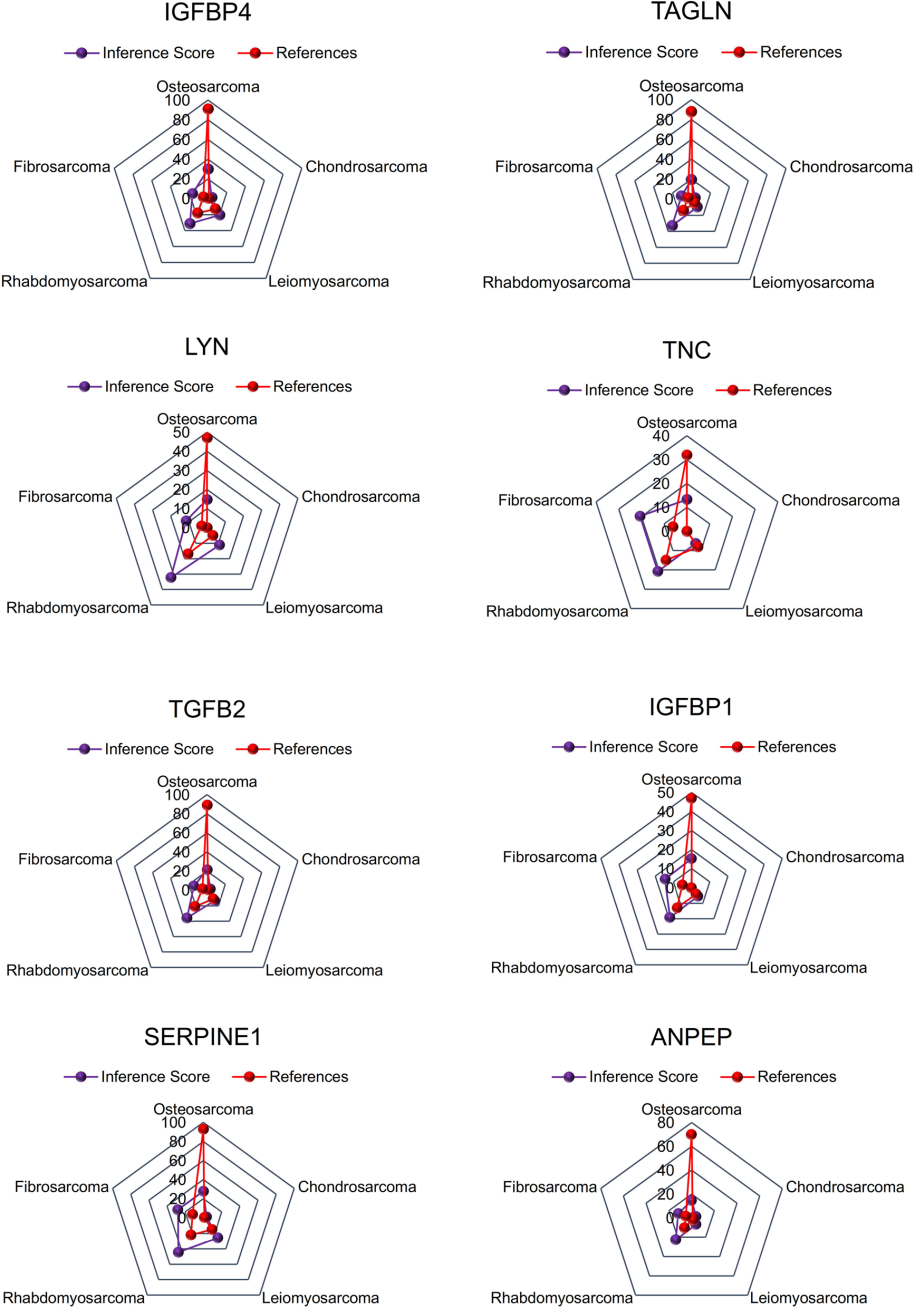
Identification of inference score of hub genes in the osteosarcoma by the CTD database.

The CTD database showed that significant hub genes targeted osteosarcoma and the data was showed in Fig. 6. There existed strong value of IGFBP4 and TAGLN on the development and occurrence of osteosarcoma.

### Enrichment analysis by GSEA

**Figure 7:**
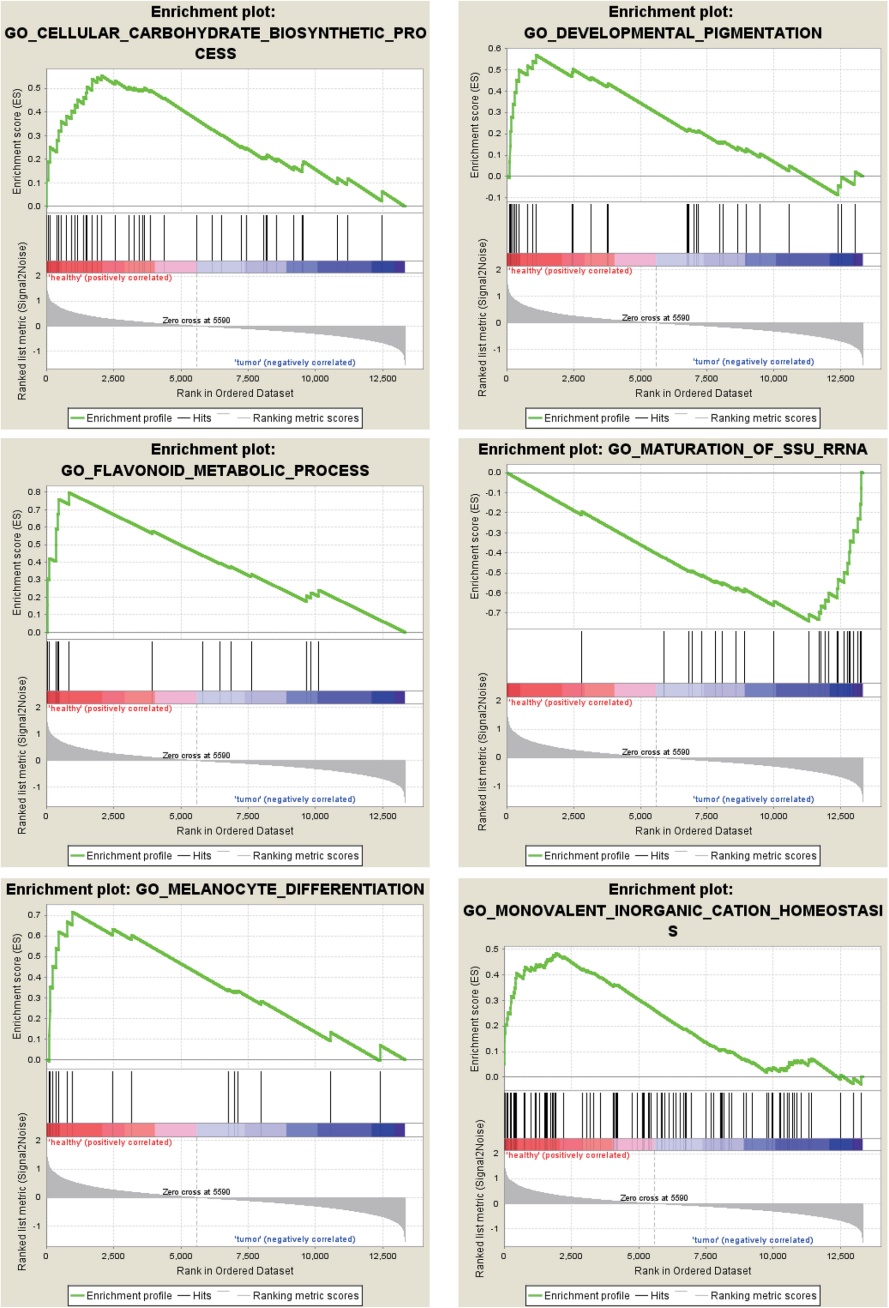
GO enrichment analysis for the DEGs by GSEA.

The most significant GO enrichments for gene sets of osteosarcoma in the significant order were “CELLULAR CARBOHYDRATE BIOSYNTHETIC PROCESS”, “DEVELOPMENTAL PIGMENTATION”, “FLAVONDIO METABOLIC PROCESS”, “MATURATION OF SSU RRNA”, “MELANOCYTE DIFFERENTIATION”, “MONOVALENT INORGANIC CATION HOMEOSTASI” (Fig. 7).

### The protein expression of IGFBP4 and TAGLN in the osteosarcoma

**Figure 8:**
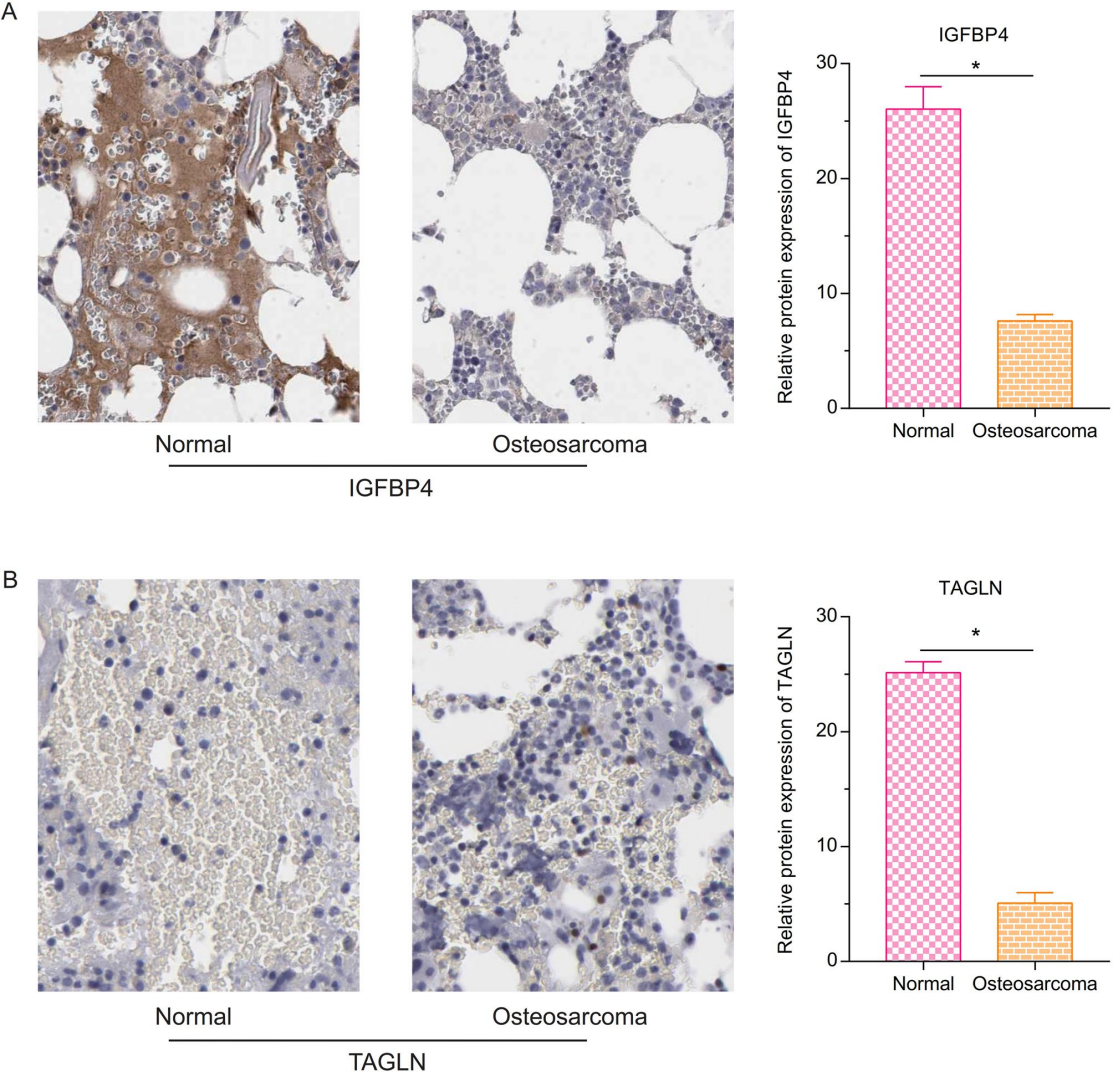
The protein expression of IGFBP4 and TAGLN in the osteosarcoma by the Human Protein Atlas. (**A**) Protein expression of IGFBP4 in the osteosarcoma was lower than the normal. (**B**) The protein expression of TAGLN in the osteosarcoma was lower than the normal (*P < 0.05).

By the Human Protein Atlas, protein expression of IGFBP4 (Fig. 8A) and TAGLN (Fig. 8B) in the osteosarcoma was lower than the normal (P < 0.05).

### Associations between characteristics, IGFBP4 and TAGLN based on χ^2^ test

There were 54 male patients and 80 female individuals. In addition, all patients included 65 cases with age < 65 years old and 69 cases with age ≥ 65 years old. And there were 60 individuals with tumor size < 5 cm and 74 cases with tumor size ≥ 5 cm. The number of patients with pathologic grade I was 45, II was 43, III was 46. A total of 88 cases came from primary tumors, and 46 patients were of tumor metastasis. The number of patients with Ennenking stage I, II, III, and IV were 25, 38, 33, and 38.

Pearson's Chi-square test results showed the correlation between IGFBP4 and relevant clinical factors. In the individuals, pathologic grade (P = 0.017), tumor metastasis (P < 0.001), and enneking stage (P < 0.001) were significantly correlated with IGFBP4. However, sex (P = 0.441), age (P = 0.094), tumor size (P = 0.649) had no significant correlation with IGFBP4.

The results showed that pathologic grade (P = 0.002), tumor metastasis (P < 0.001), and enneking stage (P < 0.001) were markedly related to the TAGLN. However, there was no effective correlation between TAGLN and sex (P = 0.277), age (P = 0.183), tumor size (P = 0.629) (Table 1).

**Table 1:** Clinicopathological variables and the expression status of IGFBP4 and TAGLN.

		IGFBP4			P	TAGLN			P
		−/+ (%)	++ (%)	+++ (%)		−/+ (%)	++ (%)	+++ (%)	
**Sex**									
Male	54	18 (13.4%)	15 (11.2%)	21 (15.7%)	0.441	16 (11.9%)	24 (17.9%)	14 (10.4%)	0.277
Female	80	21 (15.7%)	19 (14.2%)	40 (29.9%)		21 (15.7%)	27 (20.1%)	32 (23.9%)	
**Age**									
< 65 years	65	24 (17.9%)	17 (12.7%)	24 (17.9%)	0.094	22 (16.4%)	25 (18.7%)	18 (13.4%)	0.183
≥ 65 years	69	15 (11.2%)	17 (12.7%)	37 (27.6%)		15 (11.2%)	26 (19.4%)	28 (20.9%)	
**Tumor size**									
< 5 cm	60	19 (14.2%)	13 (9.7%)	28 (20.9%)	0.649	19 (14.2%)	22 (16.4%)	19 (14.2%)	0.629
≥ 5 cm	74	20 (14.9%)	21 (15.7%)	33 (24.6%)		18 (13.4%)	29 (21.6%)	27 (20.1%)	
Pathologic grade*									
I	45	20 (14.9%)	10 (7.5%)	15 (11.2%)	0.017*	21 (15.7%)	13 (9.7%)	11 (8.2%)	0.002*
II	43	5 (3.7%)	12 (9.0%)	26 (19.4%)		8 (6.0%)	14 (10.4%)	21 (15.7%)	
III	46	14 (10.4%)	12 (9.0%)	20 (14.9%)		8 (6.0%)	24 (17.9%)	14 (10.4%)	
Tumor metastasis*									
No	88	0 (0.0%)	27 (20.1%)	61 (45.5%)	< 0.001*	5 (3.7%)	38 (28.4%)	45 (33.6%)	< 0.001*
Yes	46	39 (29.1%)	7 (5.2%)	0 (0.0%)		32 (23.9%)	13 (9.7%)	1 (0.7%)	
Ennenking stage*									
I	25	1 (0.7%)	4 (3.0%)	20 (14.9%)	< 0.001*	1 (0.7%)	3 (2.2%)	21 (15.7%)	< 0.001*
II	38	4 (3.0%)	7 (5.2%)	27 (20.1%)		3 (2.2%)	15 (11.2%)	20 (14.9%)	
III	33	17 (12.7%)	11 (8.2%)	5 (3.7%)		18 (13.4%)	11 (8.2%)	4 (3.0%)	
IV	38	17 (12.7%)	12 (9.0%)	9 (6.7%)		15 (11.2%)	22(16.4%)	1(0.7%)	

Pearson’s Chi-squared test was used. *P < 0.05.

### Spearman's correlation test was used to determine further the correlation between potential characteristics, IGFBP4 and TAGLN

Spearman’s correlation test showed that IGFBP4 were significantly correlated with the tumor metastasis (ρ = − 0.843, P < 0.001), enneking stage (ρ = − 0.500, P < 0.001), and TAGLN (ρ = 0.821, P < 0.001). However, other related parameters had no significant correlation with IGFBP4.

Spearman’s correlation coefficient displayed that pathologic grade (ρ = 0.177, P = 0.041), tumor metastasis (ρ = − 0.677, P < 0.001), and enneking stage (ρ = − 0.600, P < 0.001) were significantly correlated with TAGLN. However, the other related parameters are not significantly related to TAGLN (Table 2).

**Table 2:** The corelationship between characteristics of patients and IGFBP4 and TAGLN.

Characteristics	IGFBP4		TAGLN	
	ρ	P (spearman)	ρ	P (spearman)
Sex	0.107	0.218	0.112	0.197
Age*	0.187	0.030*	0.159	0.066
Tumor size	0.010	0.910	0.076	0.382
Pathologic grade	0.111	0.200	0.177	0.041*
Tumor metastasis*	− 0.843	< 0.001*	− 0.677	< 0.001*
Ennenking stage*	− 0.500	< 0.001*	− 0.600	< 0.001*
TAGLN*	0.821	< 0.001*	1	< 0.001*

Spearman-rho test was used. *P < 0.05.

### Univariate and multivariate cox regression for the proportional hazards analysis of correlative factors

For overall survival, patients with tumor metastasis had higher hazard ratio (HR = 8.861, 95% CI 5.483–14.323, P < 0.001) than patients without tumor metastasis. The HR for OS was 1.542 (95% CI 0.836–2.846, P = 0.165) in the factor of enneking stage with type II compared with type I. Type III had higher HR of 3.855 (95% CI 2.050–7.248, P < 0.001) than type I. Type IV had higher HR of 4.784 (95% CI 2.538–9.021, P < 0.001) than type I. Subjects who had IGFBP4 high level was significantly better than low and medium level of IGFBP4 in OS, and the HR is 0.019 (95% CI 0.009–0.041, P < 0.001). Subjects with high TAGLN levels had significantly better OS than subjects with low and moderate TAGLN levels, and the HR is 0.010 (95% CI 0.004–0.022, P < 0.001). However, sex (HR = 0.873, 95% CI 0.577–1.321, P = 0.521), age (HR = 0.709, 95% CI 0.478–1.051, P = 0.087), tumor size (HR = 1.066, 95% CI 0.718–1.582, P = 0.752) and pathologic grade had no disadvantageous for OS significantly.

In order to control the effect of confounding factors, all factors were incorporated into the multivariate cox regression model contemporaneously. Multivariate Cox proportional regression analysis showed that IGFBP4 (HR = 0.252, 95% CI 0.122–0.517, P < 0.001) and TAGLN (HR = 0.155, 95% CI 0.089–0.269, P < 0.001) were significantly correlated with OS, whereas the sex (HR = 1.177, 95% CI 0.752–1.844, P = 0.476), age (HR = 0.894, 95% CI 0.585–1.365, P = 0.603), tumor size (HR = 1.165, 95% CI 0.776–1.750, P = 0.462), pathologic grade (HR = 1.012, 95% CI 0.791–1.295, P = 0.923), tumor metastasis (HR = 1.475, 95% CI 0.596–3.651, P = 0.400) and enneking stage (HR = 1.238, 95% CI 0.979–1.565, P = 0.075) have no correlation with OS (Table 3).

**Table 3:** Characteristics and their effect on OS based on univariate and multivariate Cox proportional regression analysis.

Characteristics		OS via the univariate regression			OS via the multivariate regression		
		HR	95% CI	P	HR	95% CI	P
**Sex**							
Male	54	1		0.521	1.177	0.752–1.844	0.476
Female	80	0.873	0.577–1.321				
**Age**							
< 65 years	65	1		0.087	0.894	0.585–1.365	0.603
≥ 65 years	69	0.709	0.478–1.051				
**Tumor size**							
< 5 cm	60	1		0.752	1.165	0.776–1.750	0.462
≥ 5 cm	74	1.066	0.718–1.582				
**Pathologic grade**							
I	45	1			1.012	0.791–1.295	0.923
II	43	0.647	0.395–1.060	0.084			
III	46	0.782	0.494–1.238	0.294			
Tumor metastasis*							
No	88	1			1.475	0.596–3.651	0.400
Yes	46	8.861	5.483–14.323	< 0.001*			
Ennenking stage*							
I	25	1			1.238	0.979–1.565	0.075
II	38	1.542	0.836–2.846	0.165			
III	33	3.855	2.050–7.248	< 0.001*			
IV	38	4.784	2.538–9.021	< 0.001*			
IGFBP4*							
Low (−/+)		1			0.252	0.122–0.517	< 0.001*
Moderate (++)		0.108	0.056–0.208	< 0.001*			
High (+++)		0.019	0.009–0.041	< 0.001*			
TAGLN*							
Low (−/+)		1			0.155	0.089–0.269	< 0.001*
Moderate (++)		0.096	0.052–0.175	< 0.001*			
High (+++)		0.010	0.004–0.022	< 0.001*			

OS, overall survival; HR, hazard ratio; 95% CI, 95% confidence interval. * P < 0.05.

### The expression of IGFBP4 and TAGLN were detected by RT-PCR

**Figure 9:**
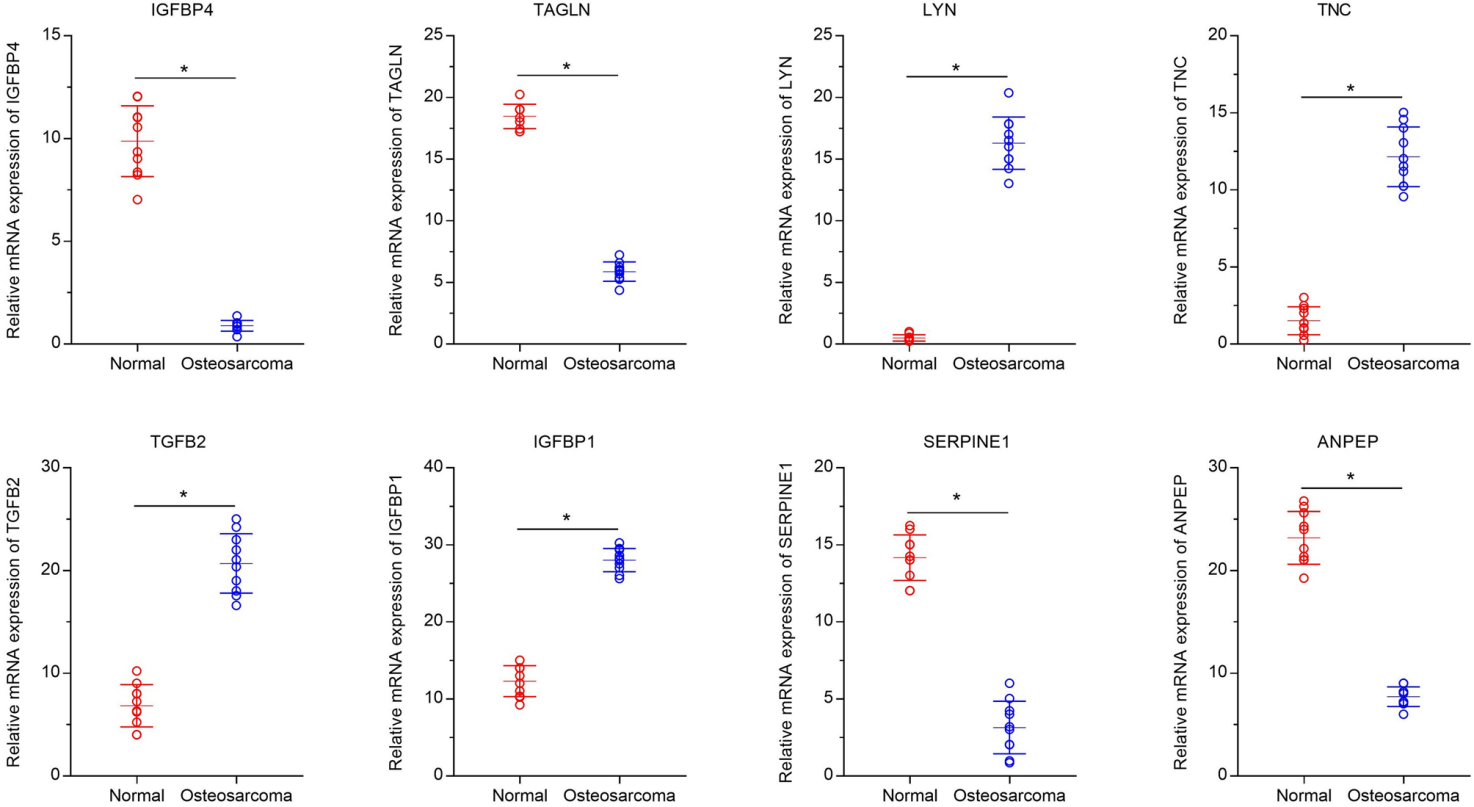
RT-qPCR for hub genes. The relative expression levels of IGFBP4, SERPINE1, ANPEP and TAGLN were significantly lower in the osteosarcoma group compared with the normal group. However, the relative expression levels of LYN, TNC, TGFB2, and IGFBP1 were significantly higher in the osteosarcoma group compared with the normal group. (*P < 0.05).

The relative expression levels of IGFBP4, SERPINE1, ANPEP and TAGLN were significantly lower in the osteosarcoma group compared with the normal group. However, the relative expression levels of LYN, TNC, TGFB2, and IGFBP1 were significantly higher in the osteosarcoma group compared with the normal group (P < 0.05, Fig. 9).

### The low expression of IGFBP4 and TAGLN in the osteosarcoma via the immunofluorescence

**Figure 10:**
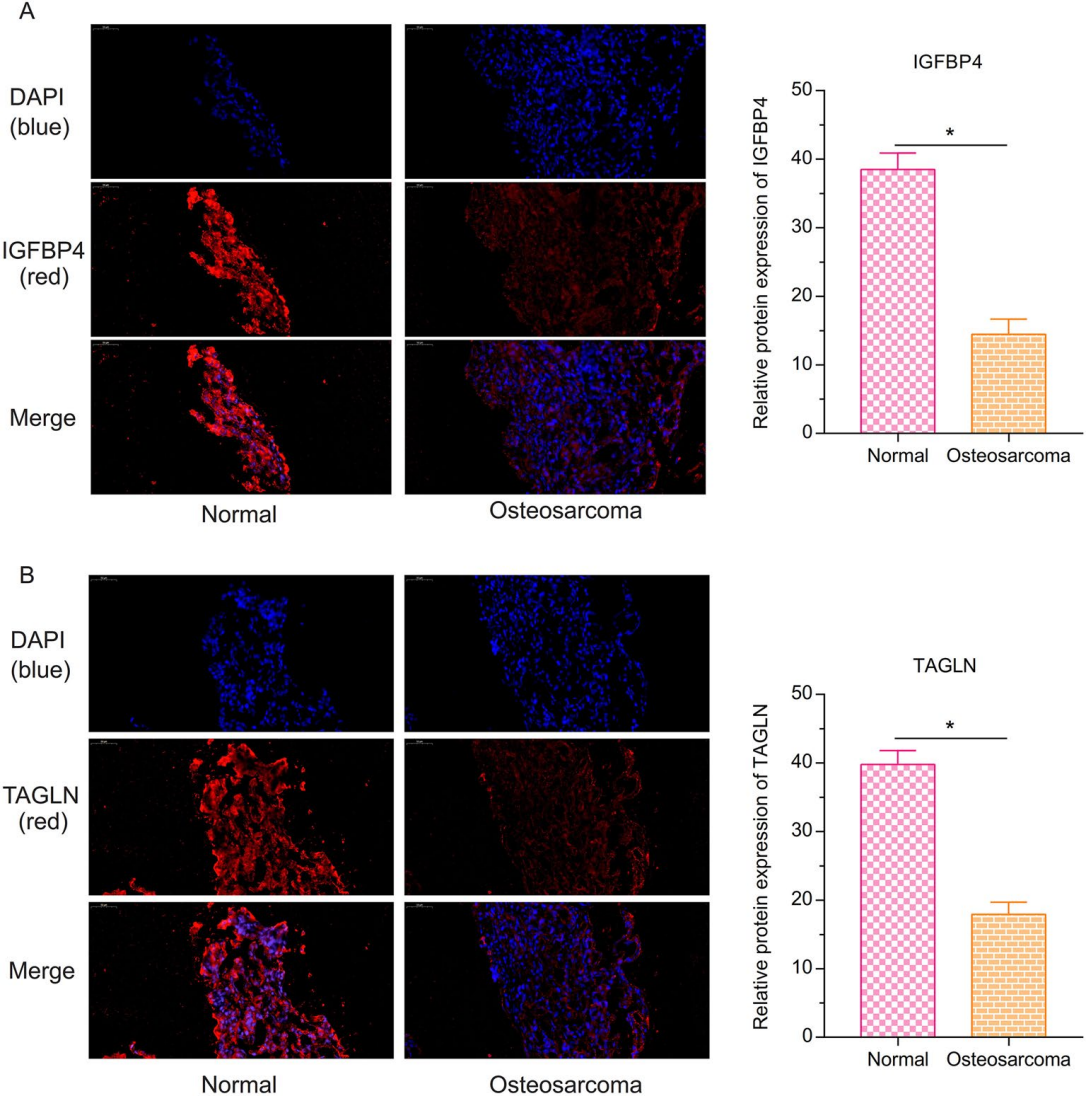
The verification of protein expression of IGFBP4 and TAGLN by the immunofluorescence assay. Protein expression of (**A**) IGFBP4 and (**B**) TAGLN in the osteosarcoma was lower than the normal. (*P < 0.05).

In the immunofluorescence assay, the blue color represents the nucleus, and the red represents the target gene. Compared with the normal group, the relative expression of IGFBP4 (Fig. 10A) and TAGLN (Fig. 10B) in the osteosarcoma animal model was lower (P < 0.05).

### Verification the predictable value of IGFBP4 and TAGLN for the osteosarcoma via the support vector machine (SVM)

**Figure 11:**
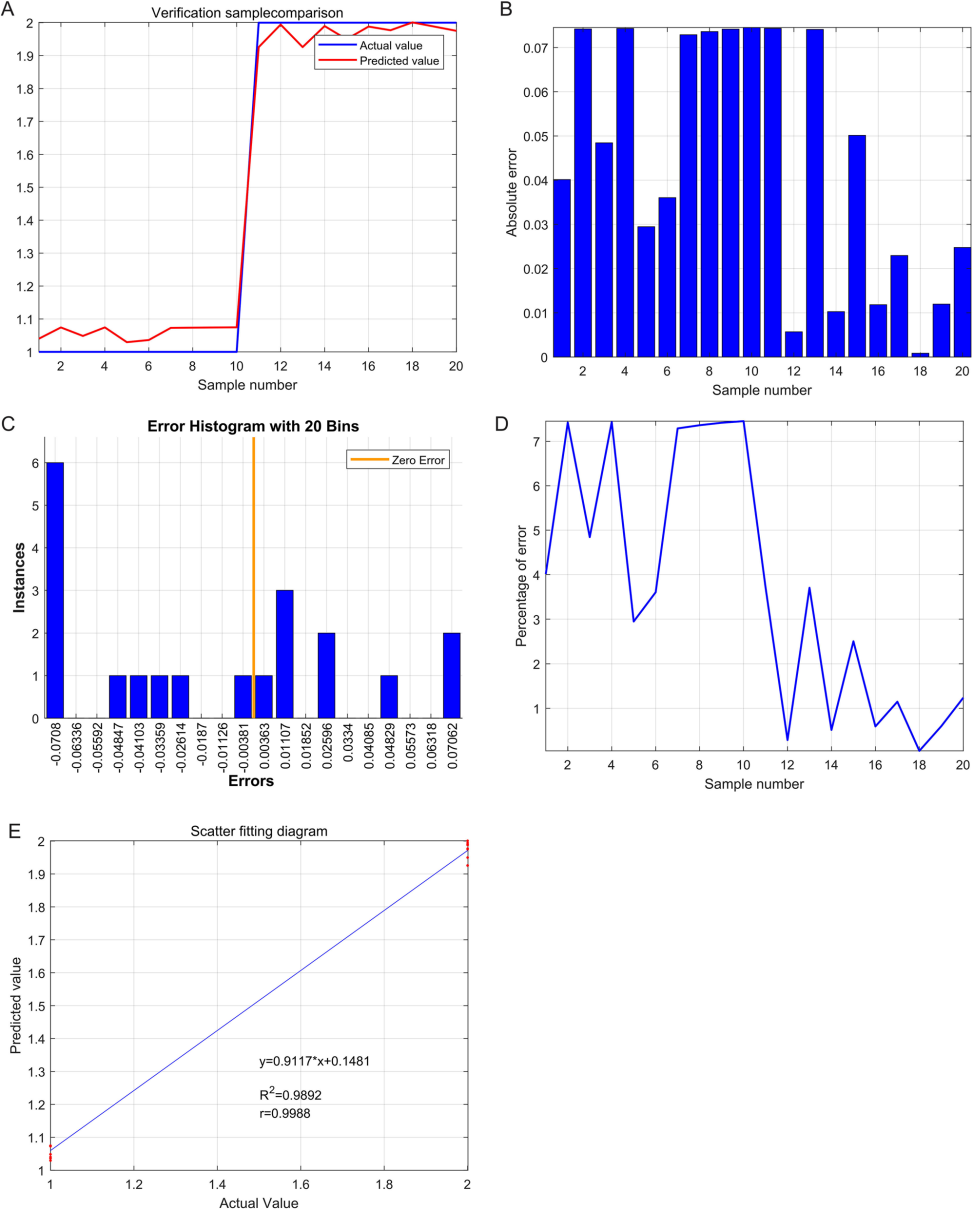
Verification the strong predictable value of IGFBP4 and TAGLN for the osteosarcoma via the support vector machine (SVM). (**A**) Verification sample comparison. (**B**) Absolute error (**C**) Error histogram with 20 bins. (**D**) Percentage of error. (**E**) In the scatter fitting diagram, the relationship between the predicted value and the actual value is that: y = 0.9117*x + 0.1481, R^2^ = 0.9892, r = 0.9988.

In the verification sample comparison, the trend of actual value was similar with the predicted value via the SVM model (Fig. 11A). Absolute error was less than the 0.08 (Fig. 11B). The error histogram with 20 bins was closed title the zero error (Fig. 11C). Furthermore, the percentage of error was less than the 7% (Fig. 11D). In the scatter fitting diagram, the relationship between the predicted value and the actual value is that: y = 0.9117*x + 0.1481, R^2^ = 0.9892, r = 0.9988 (Fig. 11E).

## Discussion

This study used bioinformatics techniques to analyze normal cells’ osteosarcoma and screen hub genes. It was found that the IGFBP4 gene and TAGLN gene were under-expressed in osteosarcoma, and when the two genes were under-expressed, patients had a poor prognosis.

Most studies suggest that the function of the human IGFBP4 gene is similar to that of tumor suppressor genes^21^. In recent years, studies have shown that IGFBP4 can play an essential role in regulating the growth of various tumor cells through IGFs-dependent or IGFs-independent mechanisms^6,22^. Lee et al.^6^ found that the absence of tumor suppressor IGFBP4 promotes hepatocellular carcinoma. Yang et al.^23^ found that the overexpression of lncRNA IGFBP4-1 reprograms energy metabolism, thus enabling lung cancer progression. lnc-IGFBP4-1 is significantly up-regulated in lung cancer tissues and plays an active role in cell proliferation and metastasis through the possible mechanism of reprogramming tumor cell energy metabolism. Ryan et al.^24^ found that the expression of a protease-resistant IGFBP4 inhibits tumor growth in a murine model of breast cancer. MeCP2 plays a vital role in the proliferation, migration, and invasion of osteosarcoma^25^. Meng et al.^26^ screened out 5 DEGs related to the MeCP2 gene through gene microarray analysis, including IGFBP4, HOXC8, LMO4, MDK, and CTGF. It might have participated in propagating osteosarcoma cells mediated by the MeCP2 gene. In this study, the IGFBP4 gene was low expressed in osteosarcoma, and low expression had a poor prognosis.

TAGLN is mainly expressed in fibroblasts and smooth muscle cells and is located in cytoskeletal organs. It stimulates actin cross-linking and participates in cytoskeletal remodeling under certain conditions, and cytoskeletal structure and function alterations are intimately associated with tumor cell production and migration^27^. Furthermore, TAGLN is implicated in extracellular matrix disintegration and angiogenesis in smooth muscle development into stem cells and embryonic blood vessels, contributing to tumor cell invasion and angiogenesis. Wu et al.^28^ found that TAGLN expression in lung adenocarcinoma cells under hypoxia conditions can promote the migration of cancer cells. Relevant studies have shown that TAGLN expression is significantly lower in the bladder, renal cell, and colorectal cancer tissues than the corresponding normal tissues^29,30^. Li et al.^31^ found that the overexpression of TAGLN could reduce the proliferation and invasion of colorectal cancer cells, which supported the role of TAGLN as a tumor suppressor. Zhao et al.^32^ found that TAGLN is a direct target of miR-144 in osteosarcoma and indicate that miR-144 exerts its anti-metastatic effects by inhibiting TAGLN expression. In this study, the TAGLN gene was low expressed in osteosarcoma, and low expression had a poor prognosis, which was the same as previous studies.

### Deficiency and prospects

Although rigorous bioinformatics analysis was performed in this paper, there are still some shortcomings. In this study, no animal experiments were conducted for over-expression or knockout to verify the function further. This study showed that IGFBP4 and TAGLN were of low expression in patients with osteosarcoma. The results showed that patients with under-expression of IGFBP4 and TAGLN had a poor prognosis, consistent with the results reported in previous literature. IGFBP4 and TAGLN might be the inhibitor of osteosarcoma. Furthermore, predictable value of IGFBP4 and TAGLN for the osteosarcoma was found via the support vector machine (SVM).

Finally, IGFBP4 and TAGLN may be attractive molecular targets for osteosarcoma, opening a new avenue for research into the disease.

## Data Availability

The datasets used and/or analyzed during the current study are available from the corresponding author on reasonable request.

## Abbreviations

BP: Biological process
CC: Cellular component
MF: Molecular function
GEO: Gene Expression Omnibus
OS: Overall survival
MCODE: Molecular Complex Detection tool
HRs: Hazard ratios
95% CI: 95% Confidence intervals
LDP: Lactate dehydrogenase
